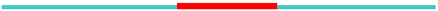# Trends and New Developments in *Artemia* Research

**DOI:** 10.3390/ani12182321

**Published:** 2022-09-07

**Authors:** Mohamad Nor Azra, Mohd Iqbal Mohd Noor, Juris Burlakovs, Muhammad Fuad Abdullah, Zulkiflee Abd Latif, Yeong Yik Sung

**Affiliations:** 1 Institute of Marine Biotechnology (IMB), Universiti Malaysia Terengganu (UMT), Kuala Terengganu 21030, Terengganu, Malaysia; 2 Climate Change Adaptation Laboratory, Institute of Marine Biotechnology (IMB), Universiti Malaysia Terengganu (UMT), Kuala Nerus 21030, Terengganu, Malaysia; 3 Institute for Biodiversity and Sustainable Development, Universiti Teknologi MARA (UiTM), Shah Alam 40450, Selangor, Malaysia; 4 Faculty of Business Management, Universiti Teknologi MARA (UiTM) (Pahang), Raub 27600, Pahang, Malaysia; 5 The Mineral and Energy Economy Research Institute of the Polish Academy of Sciences, Wybickiego 7A, 31-261 Krakow, Poland

**Keywords:** crustacea, decapoda, fish, larval rearing, shrimp

## Abstract

**Simple Summary:**

*Artemia* is an important crustacean group, especially for aquaculture live food and as a model organism for toxicity assessment. The present study aimed to identify the current trends, research gaps, and literature development in the study of *Artemia* around the world. This primitive *Arthropod* has undergone significant evolution in terms of its application in various industries as well as relevant literature patterns in terms of scientometric analyses.

**Abstract:**

An increasing number of scientists since 1970 has examined *Artemia* as an important species in aquaculture-related fields. However, a global scientometric review of *Artemia* literature is still lacking, which is the objective of this research. Using a CiteSpace analysis, the distribution of core authors and institutions, highly cited keywords and papers, author and journal contributions, and hot topics in the literature, as well as a co-citation analysis, particularly regarding authors, journals, documents, and clusters, were determined. Hence, 8741 relevant publications were generated from the Web of Science Core Collection database. The results revealed that the most significant contributions in Artemia research primarily originated from the USA, Brazil, Spain, India, China, and Belgium. Moreover, *Artemia* research focused mainly on top keywords such as brine shrimp and antimicrobial activity. Emerging trends related to *Artemia* research were Atlantic halibut, elongation factor, *Artemia salina*, lean protein, inert diet, alpha-crystallin protein, and *Artemia* embryo. At the same time, the study generated a vast total of 45 co-citation clusters. The present study provides the existing body of knowledge on *Artemia* research by sharing a visual knowledge map. This study offers a valuable perspective and profound understanding for researchers, farmers, and consortia interested in promoting *Artemia* as a sustainable live food in the global aquaculture industry.

## 1. Introduction

*Artemia*, or brine shrimp, is a primitive group of *Arthropods* with sizes between 8 and 12 mm. They are endemic to Asia, Europe, Africa, and Australia. They are sexually reproducing or bisexual species; few are parthenogenetically reproducing populations [1]. Historically, *Artemia* was considered a potential economic commodity species as early as the 1930s, and it was commercially marketed around the 1950s. Naturally, the Great Salt Lake in the USA was the first available natural habitat for the *Artemia*. However, because of their increasing demand for marine fish and crustacean larviculture [2], many inland salt lakes have emerged, such as in Bohai Bay, China, southern Siberia, Kazakhstan, and seasonal solar saltworks in the Mekong Delta, Vietnam. With the help of the European Union budget through the WorldFish organization, new efforts emerged for the potential sustainable production of *Artemia* in the area at Cox’s Bazar District, located in the southeast coastal zone of Bangladesh, through their available solar salt farm technology [3].

*Artemia* has been studied across various fields, for example, considering the ability of *Artemia* seed to produce feed for fish or shellfish species [4,5], as a potential model organism for toxicity-related assessments [6,7], as a potential filter feeder [8], and as a potential candidate for determining the effects of various environmental factors [9]. An improvement has also been made to increase the ability of *Artemia* as an exogenous feed for various fish or crustacean larviculture [10], as well as their optimal cultivation in various culture conditions [11,12]. 

As *Artemia* has been used widely in the aquaculture industry, it is vital to keep abreast of emerging patterns and to develop collective knowledge. In recent years, scientometrics has become essential for assessing and analyzing research as well as developing selected themes or topics from science to social sciences [13]. Various tools, such as VOSviewer and CiteSpace, can be applied to determine the various subject areas of current research and development [14].

An increasing amount of the literature since 1970 has examined *Artemia* as a critical species in aquaculture-related fields. However, a global scientometric review of *Artemia-*related literature is still lacking. Thus, this research’s main objective is to determine the research gaps and bridge them for future references. Through a CiteSpace analysis, the distribution of core authors and institutions, highly cited keywords and papers, author and journal contributions, and hot topics of the literature, as well as a co-citation analysis, particularly regarding authors, journals, documents, and clusters, are determined.

This study is expected to offer a valuable perspective and in-depth understanding to researchers, farmers, and consortia who wish to promote *Artemia* as a sustainable live food in the aquaculture industry in the world.

## 2. Survey Methods

**Figure 1:**
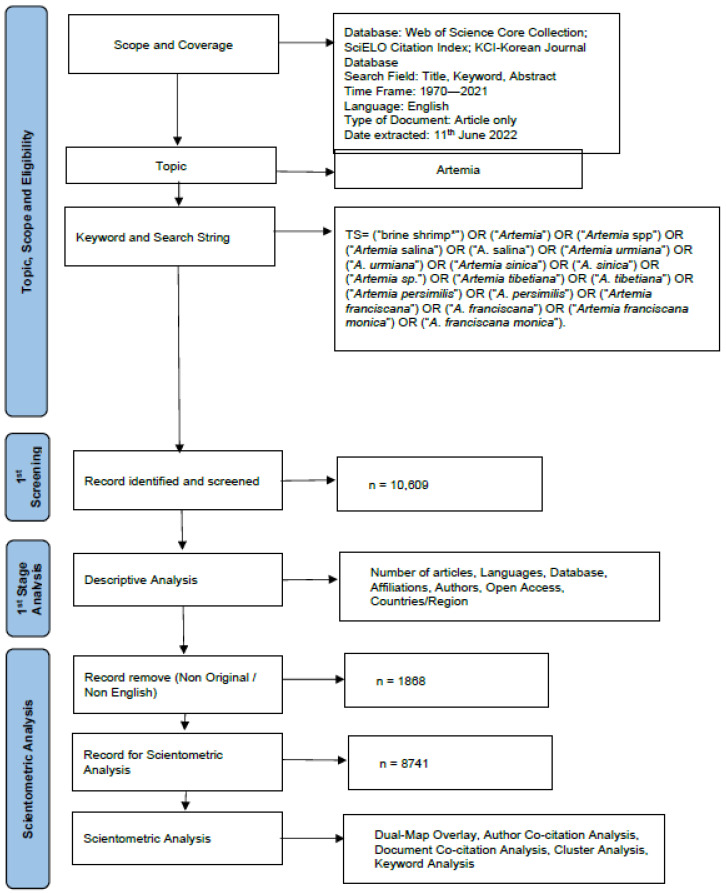
Flowchart for research structure on *Artemia* literature in the world focused on the Web of Science Core Collection (WOSCC) database.

The details of the research framework used in this study are shown in Figure 1. The survey methods include a description of the metadata search and a scientometrics-based analysis. To conduct our scientometric analysis, we followed the protocol outlined by Chen [15].

### 2.1. Data Sources

The database from Web of Science (WOS) is used to administer a manuscript search for a scientometric review. We concentrated on the navigation of the entire citation network of the WOSCC database of subscription materials, which is regarded as one of the top databases of scholarly journals, books, and proceedings in the sciences, social sciences, and arts as well as humanities in the world. Major bibliographic sources, such as WOS, routinely include cited references, whereas other sources, such as PubMed and China National Knowledge Infrastructure (CNKI), do not include cited references [15]. Additionally, for these reasons and because of its wide coverage of different disciplines, as stated above, we focused on using WOS to retrieve scientific literature.

### 2.2. Article Search

The “subject” (TS) field served as the basis for the WOS article searches, which included article titles, keywords, abstracts, and “KeyWords Plus”. The keyword “Plus” is a generated term pulled automatically from the titles of cited articles in the WOS database. *Artemia* species’ common names and available scientific names were based on the Food and Agriculture Organization of the United Nations (FAO) catalog published on their respective website or referred to on their “Cultured Aquatic Species Information Programme’’ for the *Artemia* group [1]. We used two tags in WOS: the Boolean function (OR) and asterisk symbols (*). The asterisk symbols were applied to identify variations in the keywords available in the WOS database. In order to identify articles related to *Artemia*, the following key terms were used:

*Artemia*: (“brine shrimp*”) OR (“*Artemia*”) OR (“*Artemia* spp.”) OR (“*Artemia salina*”) OR (“A. salina”) OR (“*Artemia urmiana*”) OR (“*A. urmiana*”) OR (“*Artemia sinica*”) OR (“*A. sinica*”) OR (“*Artemia* sp.”) OR (“*Artemia tibetiana*”) OR (“*A. tibetiana*”) OR (“*Artemia persimilis*”) OR (“*A. persimilis*”) OR (“*Artemia franciscana*”) OR (“*A. franciscana*”) OR (“*Artemia franciscana monica*”) OR (“*A. franciscana monica*”).

### 2.3. Eligibility Criteria

Several eligibility criteria are required for conducting the scientometric review, especially during the scientific literature search. Firstly, the research articles are original research papers written in English. In addition, we exclude studies in the form of conference papers, chapters in a book, review papers, letters, editorials, and abstracts. Such articles were excluded because they might complicate the analytical process, since they might add any tangible value to the analyzed results. However, our results may include books and chapters within a book because the co-citation analysis automatically includes books/chapters when they are cited by original research articles.

### 2.4. Data and Scientometric Analysis

CiteSpace was used to analyze the metadata for the present study, which will support the data mining from WOS through the visual summarization features of *Artemia* research. The present study used CiteSpace V version 6.1.R2, Basic (built 6/20/2022; expires 31 December 2022) for 64-bit Windows along with Microsoft Office Professional Plus 2019, the Excel spreadsheet software (i.e., apps). A co-citation instance occurs when two sources are cited in a single work, and a co-citation analysis of journals, authors, and articles was determined to acquire a cluster of co-citing factors [16,17,18]. A variable’s quality was apprised using degree, centrality, and sigma [15].

## 3. Results

### 3.1. Descriptive Statistics

#### 3.1.1. Total Number of Articles

**Figure 2:**
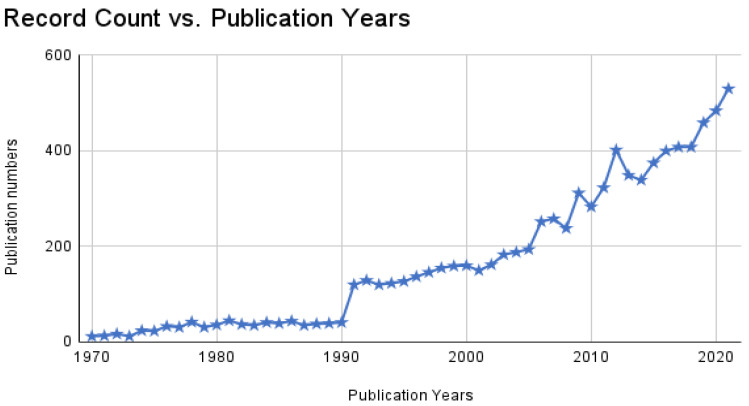
The number of research articles published annually since 1970.

Our analysis focused on scientific articles published between January 1970 and December 2021. During this time, 8741 articles were generated, and the total number of articles increased from 12 in 1970 to 530 in 2021. The number of papers published rose annually, with a 10-year average of 1714 (8741 pieces/5.1 decades of publishing growth) (Figure 2).

#### 3.1.2. Productive Authors

There was a vast number of authors involved in *Artemia* research: 23,632 authors, from a total of 51 years of publications. The average number of authors is 463 authors per year. The author with the highest number of publications is known as the “Father of *Artemia*”, Patrick Sorgeloos, from Ghent University, Belgium, with 190 related publications, followed by his colleague, Peter Bossier, with his international collaborators, and Thomas H. MacRae, from Dalhousie University, Canada, as the third most published author in *Artemia*-related research in the world, based on the WOS Core Collection database (Table 1).

#### 3.1.3. Top Institutions

**Figure 3:**
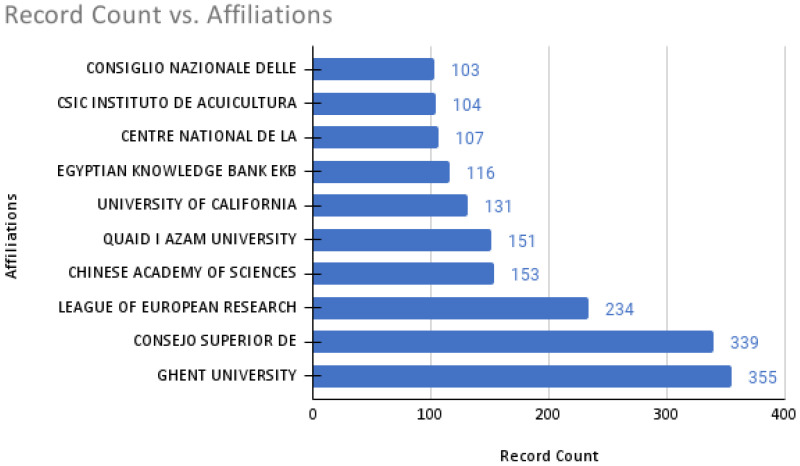
The number of manuscripts from the top ten of the 4739 institutions involved in *Artemia* research.

With 355 publications, Ghent University, Belgium, was the leading institution among a total of 4739 institutions involved in *Artemia* research worldwide (Figure 3). The Spanish National Research Council (Consejo Superior de Investigaciones Científicas), the largest public institution dedicated to research in Spain and the third largest in Europe, is the second leading institute that published a research article on *Artemia-*related studies in the world. Institutions produced 1.8 articles on average.

#### 3.1.4. Productive Journals

**Figure 4:**
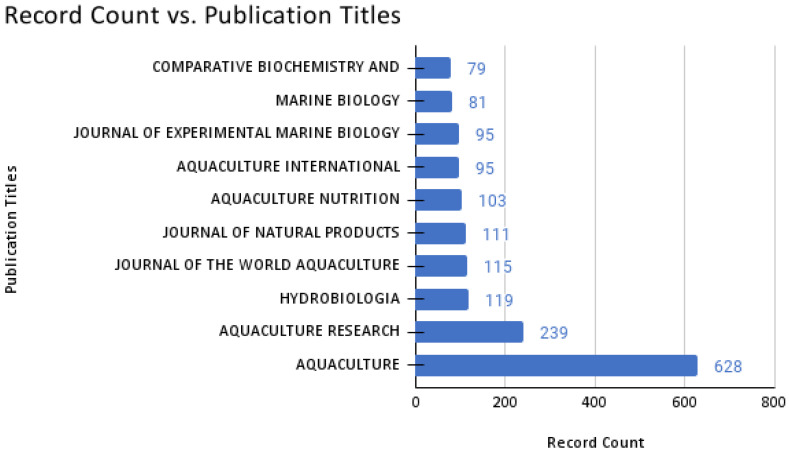
The primary or secondary sources (journals) utilized for the *Artemia-*related study literature search and scientometric analysis.

We detected eligible papers from 1554 journals in total. Of these, *Aquaculture* had the most publications (628 publications), almost triple those published by *Aquaculture Research* (239 publications), followed by *Hydrobiologia* (119 publications) (Figure 4). During the 51 years of publications, most journals released an average of 5.6 articles (1970–2021).

#### 3.1.5. Most Cited Article

This subsection indicates the most cited article in *Artemia* research in the world, based on the WOSCC database (Table 2). The results indicated that the article entitled “A review of toxicity testing protocols and endpoints with *Artemia* spp.” received the highest total number of citations, which was published in the peer-reviewed journal *Ecological Indicators* [19]. The book published by McLaughlin [20] is the second most cited scientific publication on *Artemia* research, followed by the study by Rajabi et al. [6] on the ability to use *Artemia* as a model organism for toxicity assessments. This result was based on the cluster analysis, which was automatically generated from the CiteSpace software. 

#### 3.1.6. Regional Distribution

**Figure 5:**
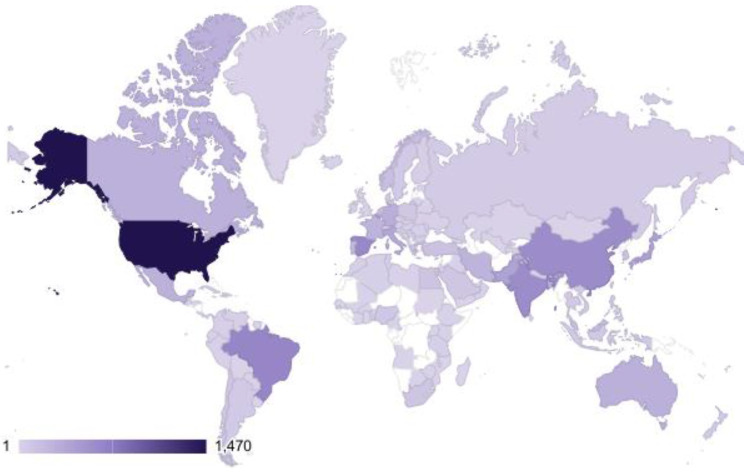
Total manuscripts per nation for *Artemia* research. The darkest shade of purple reflects the greatest number of total publications, while lighter hues imply a moderate amount to fewer publications.

Fifty-one different countries had relevant publications on *Artemia* research (Figure 5). The USA, Brazil, Spain, India, China, Belgium, Japan, Pakistan, Italy, and Germany contributed the most published articles in *Artemia* research (> 300 publications), with these top 10 countries accounting for 68.24% of the total publications. Our findings also revealed that less than a quarter of the world’s countries (51/195) are actively researching *Artemia*.

### 3.2. Scientometric

#### 3.2.1. Co-Citation Analysis

A co-citation analysis creates a science map with connections, nodes, and density values to depict the main structure of a variable’s development status as well as changes over time. The results of the co-citation analysis are shown below for authors, journals, countries/regions, institutions, and article documents.

#### 3.2.2. Author Co-Citation Analysis

**Figure 6:**
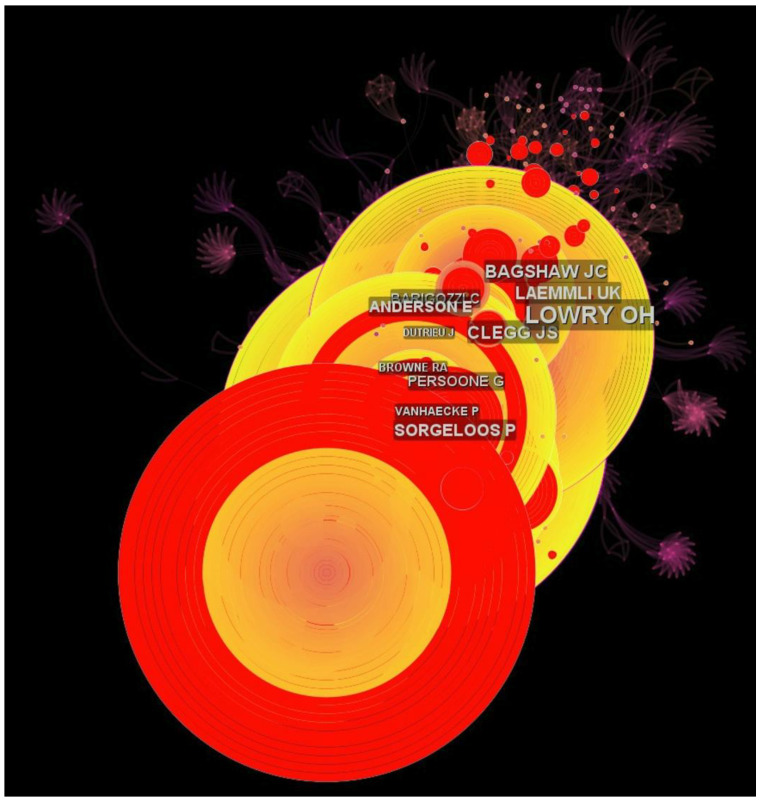
Network of authors’ co-citations, with a bigger writing format of an author’s name indicating a more cited author (more frequently referred to) in the research; the large node indicates a high number of citations (red ring), based on the WOSCC database only.

An author from Purdue University, USA, Brian N. Meyer, is the most influential author in the field, with the highest sigma score (400.34). The next most influential author, Ulrich K. Laemmli, previously from the Medical Research Council (MRC) Laboratory of Molecular Biology, is currently affiliated with the Université de Genève, Geneva, Switzerland, with a sigma score of 164.77. The third most influential author based on the sigma score was Oliver H. Lowry from Washington University, USA (Table 3). However, based on Figure 6, the most cited author is Patrick Sorgeloos, from Ghent University, Belgium.

#### 3.2.3. Journal Co-Citation Analysis

**Figure 7:**
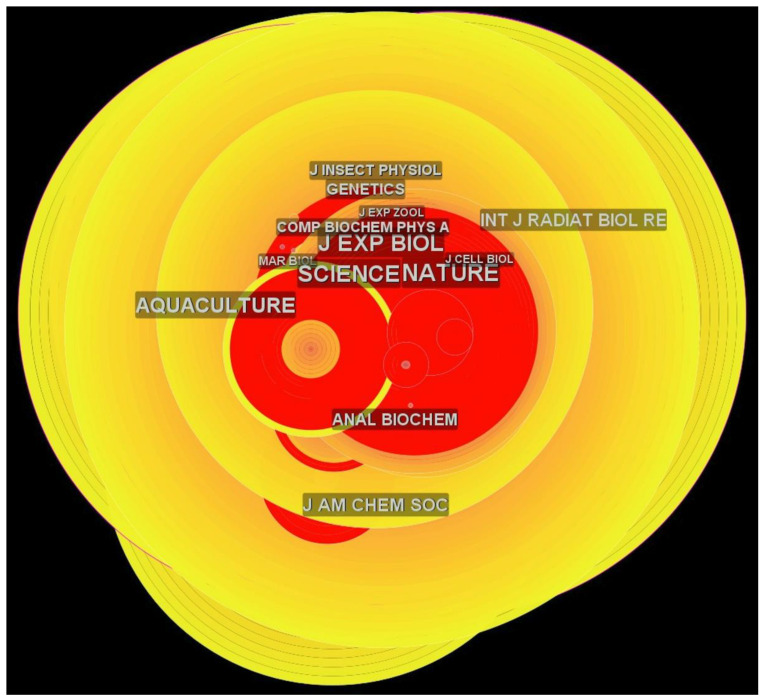
The network of journal co-citations. A journal’s name size scales with its centrality score.

Only journals with centrality scores greater than 0.1 are shown in the knowledge map of the journal co-citation analysis (Figure 7). High-impact journals, such as *Science* and *Nature*, are among the most cited journals in the field, along with *Aquaculture* and the *Journal of Experimental Biology*. Two different journals received the highest sigma score in *Artemia* research (> 90.0), which were the *Biochemical Journal* and the *Archives of Biochemistry and Biophysics* (Table 4). With a sigma score of 47.38, the *Journal of Agricultural and Food Chemistry* (IF: 5.279) was the third most influential journal.

#### 3.2.4. Document Citation Analysis

**Figure 8:**
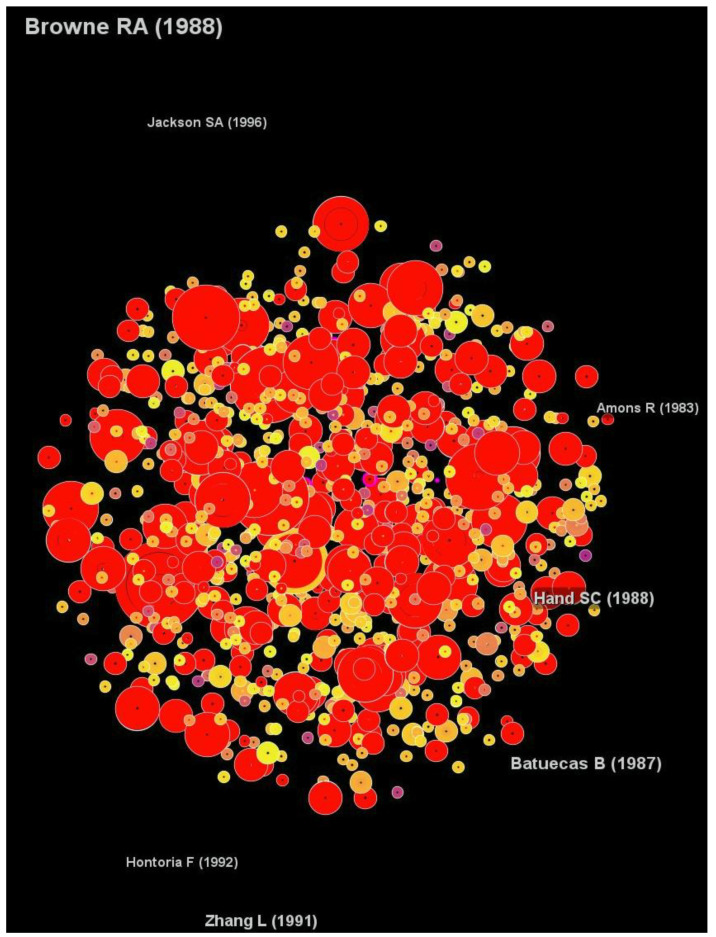
The network of the document co-citation analysis exclusively shows articles with centrality scores greater of more than 0.1.

The results only included articles with a centrality score greater than 0.1 (Figure 8). Hand and Gnaiger’s [27] article was the most influential in this field, with a sigma score of 2.14. Liang and MacRae [28] were the authors of the article with the second highest sigma value (1.96), followed by the study entitled “The American brine shrimp as an exotic invasive species in the western Mediterranean” by Amat et al. [24], with a sigma value of 1.64 (Table 5).

#### 3.2.5. Document Cluster Analysis

**Figure 9:**
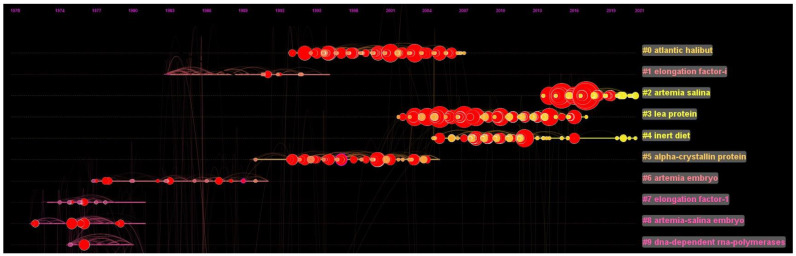
Summary of the identified top 10 document cluster lifetimes (solid lines). Cluster labels were generated from CiteSpace.

Ten significant group clusters were discovered through a document cluster analysis in the CiteSpace software, and each row in Table 6 represents a different research topic. The clusters were numbered and ranked in order of size, with #0 being the largest. Ten different clusters were summarized on a horizontal line, with the cluster label on the right side of the figure (Figure 9). The solid yellow line within each cluster represents the cluster’s lifetime. The cluster’s size is equal to the number of publications that it has. The top 10 clusters all have more than 134 publications, with cluster #0 having the greatest number of publications, 291 articles.

#### 3.2.6. Keywords’ Cluster and Burstiness Analysis

The keywords “brine shrimp”, “growth”, and “survival” were the top three highly cited keywords in *Artemia* research (Table 7), and the most popular keywords used are shown in Table 8. Typically, keyword analysis has been utilized to uncover developing trends and study hotspots. As shown in Table 9, the red line represents the burstiness period, and the blue line represents the timeline (from 1970 to 2021). We used a burst analysis to identify the most influential or landmark publications and keywords; trends among studies and keywords are described below. The greatest citation burst was “antimicrobial activity”, and this was the most vital burstiness keyword in *Artemia* research.

## 4. Discussion

Our research sought a scientometric analysis for global studies on the current trends and advancements in *Artemia-*related literature. *Artemia* has become a crucial live food for the early phase of juvenile production in the aquaculture industry, especially in marine fish culture. Additionally, *Artemia* has become a model organism for toxicity-based assessments for various chemical compositions. Thus, any research on *Artemia* could attract various early career researchers or consortia to perform or refer to *Artemia*-related studies. Our study represents the first attempt to analyze *Artemia* research publications through a scientometric analysis using the CiteSpace software. We discuss our results in detail below.

### 4.1. Evolution of the Publications

Research on *Artemia* has been published more often over the years, reaching more than 100 manuscripts per year in 1991. The trend intensified when sudden increases were also spotted at the same time between 1990 and 1991. This surge is well-known in the scientometric community because of WOS’s “artifact” effects [34]. The artifact has been caused due to the restriction of this database search only for the titles, while the searching strategies for the present studies are stated on “titles, abstracts and keywords”. Over the last five decades, a vast number of researchers has been involved in *Artemia* research, reaching almost 24,000 authors in 2021, and three out of the top ten authors mostly come from the same institution: Ghent University, Belgium. As shown by a previous animal-based scientometric analysis [35], the problems with identifying unique authors cannot be ruled out for comparison with the present study, especially for the number of authors involved in *Artemia* research. Patrick Sorgeloos was the most productive author, from Ghent University, Belgium. This achievement could be attributed to the early publication of *Artemia* studies mostly from his works and institution [36], especially on *Artemia’s* potential in the aquaculture industry. There are also many institutions involved in *Artemia* research (4739 institutions), although the number of countries does not reach 30% of the total countries in the world (Figure 5). It would also be fascinating to compare these results with other scientometric animal-based research on zebrafish. However, no data are available for the total number of affiliations involved in a relevant study [35]. Even though a total of 500 *Artemia* sites have been discovered worldwide [23], the total number of papers published based on country remains lower. Here, we strongly advocate that more international scientific research exchanges and collaboration should be conducted in the future.

The top two journals that published *Artemia* research are based on aquaculture, which are *Aquaculture* (London, UK; publisher: Elsevier) and *Aquaculture Research* (Publisher: Wiley-Blackwell Publishing Ltd.), the latter of which is the top journal that has published the highest number of articles related to *Artemia* research. Both journals support articles related to basic and applied works on the improvement of freshwater, brackish water, or marine aquaculture research and development. This fact is also supported by the aquaculture potential of *Artemia* as an early live food, mostly for commercially important aquaculture species in the world [37]. Based on the top 10 citation counts of published research manuscripts related to *Artemia* research, 40% of the articles were toxicity-related studies on *Artemia* [6,19,20,26], biology-related fields [23,24,25], aquaculture [4,21], and others [22]. The trends showed that most of the highly cited literature was related to *Artemia* as a model organism in toxicity-related studies. 

Interestingly, there are a few prominent authors who published *Artemia* research but whose work was not captured in the WOSCC database because it was published in book series [38,39,40]. Some authors excluded in this way include John A. Beardmore, Robert A. Brown, Joseph C. Bagshaw, Gonzalo Gajardo, and Theodore Abatzopoulos. All of these researchers are very popular in their field and are editors and/or corresponding authors for most *Artemia*-related research [38,39,40]. 

### 4.2. Co-Citation Trending Topic

Previous studies have shown that co-citation datasets have been used to identify collaborative networks. Most focused on the influential research of authors, journals, and documents involved in a selected theme [41]. The author co-citation analysis indicated a huge difference between the top three co-cited authors among the top ten, as listed in Table 3. The top three co-cited authors had the highest sigma scores, all more than 100. This ranking could indicate that most of their article(s) are being referred to, since the co-cited authors were analyzed using sigma scores. Authors should improve the quality of their papers and collaborate with others to increase their sigma scores and gain more citations. Surprisingly, all influential authors are researchers from the 20th (twentieth) century, which could also indicate that recently published papers in the 21st century lack collaborative research. The same is true for the journal co-citation analysis, which indicated that all journals were at their peak in the 20th (twentieth) century. The sigma score is also one of the most vital metrics for determining the most influential journal in the proposed field of research.

A “central” article serves as a mediator in the document co-citation analysis. In *Artemia* research, no article can be stated as a central article based on Figure 8. The article produced by Hand and Gnaiger [27], Liang and MacRae [28], and Amat et al. [24], according to the sigma scores, are among the top ten most influential scientific publications. Hand and Gnaiger [27] briefly explain the calorimetric test to quantify anaerobic dormancy in *Artemia* embryos. Meanwhile, the study by Liang and MacRae [28] tested the ability of a small heat shock protein, known as p26, to protect *Artemia* from thermal stress, and they found that p26 has a key role in *Artemia*’s reproduction process. Furthermore, Amat et al. [24] reviewed the distribution of *Artemia* populations in the western Mediterranean region and their origin. They also compared the impacts of *Artemia* invasive species on the available native counterparts in the region. They suggested that the aquaculture industry was introducing invasive species and that the native species in the region can rapidly replace invasive species.

In the document cluster analysis, the top three clusters that emerged in the *Artemia*-related research were Atlantic halibut, elongation factor-i, and *Artemia salina*; the most recent cluster was #2, *Artemia salina*. This analysis suggested that *Artemia salina* Leach had been used worldwide in recent years, with the important marine flatfish, Atlantic halibut, being incorporated into *Artemia* research.

The keyword with the highest count was *Artemia salina*, suggesting that this species is one of the most used research species in the field. According to experts, the most used *Artemia* species is actually *A. franciscana,* suggesting a disconnect between published science and aquaculture practices. Future research could consider the main differences between these species and why they seem to be used for different reasons. Perhaps *A. salina* is better suited for research purposes, particularly when research is not solely focused on *Artemia* but instead is secondary to the research question. The most recent keyword in the *Artemia* research was “antimicrobial activity” (strength = 23.17, 2011–2021), and the same keyword was also categorized as having the strongest citation burst among the other keywords in the field. The following top keywords were “requirement” (strength = 19.58, 1999–2008) and “decapoda” (strength = 18.99, 1997–2008).

*Artemia* is produced in every continent except for Antarctica [42,43,44,45,46,47]. Moreover, previous studies have also shown some information about the bibliometric knowledge on live feed such as *Artemia* [47], but did not focus on a detailed explanation of the studies, in contrast to the present study.

## 5. Conclusions

*Artemia* research is wide-ranging and spans many fields, yet the body of *Artemia* research focuses on two topics: its use as an aquaculture feed in fish/shellfish larviculture, and as a model organism in toxicology assessments [6,19,21,26,31]. The author who most consistently ranked highly in our qualitative and co-citation analyses was Brian N. Meyer, from Purdue University. The most influential and most well-cited author in *Artemia* research was Patrick Sorgeloos, from Ghent University, Belgium. Our results also suggested that *Artemia: Basic and Applied Biology* [48] is the most influential reference, based on the sigma score of the cluster analysis.

## 6. Limitations and Recommendations 

The current study has some limitations, including the fact that it only focused on papers from a WOS database, which could have led to publication bias and the exclusion of important field-specific works (e.g., the FAO Manual [49]). Even though our exclusion criteria should have excluded books, some book chapters were still identified in our results due to the nature of the co-citation analysis. Data downloaded from WOSCC include works that are cited by primary articles, which can help reduce some of the publication bias in our data analysis. However, it can cause some important books to still be excluded. Thus, we suggest the incorporation of additional databases that have detailed article information (e.g., titles, authors, abstracts, citations, co-citations, etc.), such as PubMed, China National Knowledge Infrastructure (CNKI), and Chinese Social Sciences Citation Index (CSSCI) [50]. We also recommend that scientometric analyses be started from early 1991 onwards to avoid any data confusion caused by the “artifact effect”, especially when searching in the WOSCC database.

## Figures and Tables

**Table 1:** Top ten most productive authors from 1970 to 2021 in *Artemia* research worldwide, based on the WOSCC database.

Author	Record Count	Affiliation
Patrick Sorgeloos	190	Ghent University
Peter Bossier	143	Ghent University
Thomas H. MacRae	84	Dalhousie University
Francisco Amat	79	Consejo Superior de Investigaciones Cientificas (CSIC)
Jerry L. Mclaughlin	71	Purdue University
Steven C. Hand	62	Louisiana State University
Gilbert Van Stappen	59	Ghent University
James S. Clegg	55	University of California Davis
Juan Carlos Navarro	52	CSIC—Instituto de Acuicultura de Torre de la Sal (IATS)
Mohan N. Patel	43	Sardar Patel University

**Table 2:** Top ten citation counts for published *Artemia*-related articles.

Article Title	Total Citations	References
A review of toxicity testing protocols and endpoints with *Artemia* spp.	45	Libralato et al. [19]
Methods in plant biochemistry: assays for bioactivity	38	McLaughlin [20]
*Artemia salina* as a model organism in toxicity assessment of nanoparticles	37	Rajabi et al. [6]
Fish larval nutrition and feed formulation: knowledge gaps and bottlenecks for advances in larval rearing	35	Hamre et al. [21]
Biostatistical analysis	29	Zarr [22]
Zoogeography	29	Stappen [23]
The American brine shrimp as an exotic invasive species in the western Mediterranean	28	Amat et al. [24]
Molecular phylogenetics and asexuality in the brine shrimp *Artemia*	28	Baxevanis et al. [25]
Use of the brine shrimp, *Artemia* spp., in marine fish larviculture	26	Sorgeloos et al. [4]
Toxicity effect of silver nanoparticles in brine shrimp *Artemia*	25	Arulvasu et al. [26]

**Table 3:** Top 10 most influential authors in *Artemia-*related research based on sigma score, analyzed from the WOSCC database.

Author	Year	Title	Degree	Centrality	Sigma
Brian N. Meyer	1990	Purdue University	31	0.06	400.34
Ulrich K. Laemmli	1976	MRC Laboratory of Molecular Biology	37	0.11	164.77
Oliver H. Lowry	1971	Washington University	46	0.16	116.29
James S. Clegg	1970	University of California	69	0.12	8.28
Joseph C. Bagshaw	1971	Worcester Polytechnic Institute	71	0.12	6.65
C. Barigozzi	1970	Università di Milano	73	0.1	6.59
Guido Persoone	1975	Ghent University	48	0.1	6.1
Gary L.Peterson	1974	University of Wisconsin	39	0.06	4.74
Takeshi Watanabe	1979	Tokyo University of Fisheries	62	0.04	3.87
E. G Bligh	1971	Canada Department of Fisheries and Environment	62	0.07	3.75

**Table 4:** The co-citation scores for the top ten journals.

Journal	Year	Impact Factor (2021)	Degree	Centrality	Sigma
*Biochemical Journal*	1970	4.097	37	0.06	123.88
*Archives of Biochemistry and Biophysics*	1970	4.013	49	0.06	90.3
*Journal of Agricultural and Food Chemistry*	1970	5.279	19	0.05	47.38
*Journal of Cell Biology*	1970	10.54	47	0.07	31.93
*Journal of Molecular Biology*	1970	5.469	35	0.04	23.67
*Developmental Biology*	1970	3.582	34	0.03	22.69
*Environmental Science & Technology*	1974	9.028	34	0.03	20.17
*Comparative Biochemistry and Physiology Part A*	1975	2.66	54	0.09	19.77
*Marine Biology*	1972	2.391	46	0.08	18.84
*Cell*	1976	41.58	34	0.03	14.75

**Table 5:** Document co-citation for the top ten articles based on the sigma score.

Title	Degree	Centrality	Sigma	Burst	Reference
Anaerobic dormancy quantified in *Artemia* embryos: a calorimetric test of the control mechanism	14	0.13	2.14	14.44	Hand and Gnaiger [27]
The synthesis of a small heat shock/α-crystallin protein in *Artemia* and its relationship to stress tolerance during development	15	0.07	1.96	11.16	Liang and MacRae [28]
The American brine shrimp as an exotic invasive species in the western Mediterranean	15	0.03	1.64	6.46	Amat et al. [24]
Morphological characterization of adult *Artemia* (*Crustacea, Branchiopoda*) from different geographical origin. Mediterranean populations	13	0.11	1.6	6.52	Hontoria and Amat [29]
Intracellular pH regulates transitions between dormancy and development of brine shrimp (*Artemia salina*) embryos	31	0.08	1.59	9.64	Busa and Crowe [30]
Fish larval nutrition and feed formulation: knowledge gaps and bottlenecks for advances in larval rearing	11	0.02	1.53	15.96	Hamre et al. [21]
Live feeds for early stages of fish rearing	18	0.03	1.51	4.34	Conceição et al. [31]
*Artemia salina* as a model organism in toxicity assessment of nanoparticles	4	0.02	1.51	5.87	Rajabi et al. [6]
Downregulation of cellular metabolism during environmental stress: mechanisms and implications	26	0.04	1.42	21.69	Hand and Hardewig [32]
Phylogeography and local endemism of the native Mediterranean brine shrimp *Artemia salina* (*Branchiopoda: Anostraca*)	14	0.03	1.37	13.04	Munoz et al. [33]

**Table 6:** The ten significant group clusters that emerged from the document co-citation analysis of *Artemia* research.

ClusterID	Size	Silhouette	Label (LLR)	Average Year	Influential Article
0	291	0.987	Atlantic halibut	2000	Characterization of protease activity in developing discus *Symphysodon aequifasciata* larva
1	216	0.974	Elongation factor-i	1986	Genes coding for the elongation factor EF-1α in *Artemia*
2	185	0.989	*Artemia salina*	2018	Acute and chronic effects of polystyrene microplastics on brine shrimp: First evidence highlighting the molecular mechanism through transcriptome analysis
3	162	0.983	Lea protein	2010	The use of a multidisciplinary approach for the characterization of a diploid parthenogenetic *Artemia* population from Torre Colimena (Apulia, Italy)
4	158	0.983	Inert diet	2010	Commercial products for *Artemia* enrichment affect growth performance, digestive system maturation, ossification and incidence of skeletal deformities in Senegalese sole (*Solea senegalensis*) larvae
5	156	0.979	Alpha-crystallin protein	1998	The heat shock response of adult *Artemia franciscana*
6	147	0.943	*Artemia* embryo	1984	Kinetic properties of hexokinase under near-physiological conditions. Relation to metabolic arrest in *Artemia* embryos during anoxia
7	145	0.95	Elongation factor-1	1976	Isolation and characterization of acidic phosphoproteins of 60-s ribosomes from *Artemia salina* and rat-liver
8	140	0.963	*Artemia salina* embryo	1975	Protein synthesis in brine shrimp embryos
9	134	0.987	DNA-dependent rna-polymerases	1977	Messenger-rna during early embryogenesis in *Artemia salina*—altered translatability and sequence complexity

**Table 7:** Top 10 clusterIDs generated from CiteSpace.

ClusterID	Size	Silhouette	Label (LLR)	Average Year
0	291	0.987	Atlantic halibut	2000
1	216	0.974	Elongation factor-i	1986
2	185	0.989	*Artemia salina*	2018
3	162	0.983	Lea protein	2010
4	158	0.983	Inert diet	2010
5	156	0.979	Alpha-crystallin protein	1998
6	147	0.943	*Artemia* embryo	1984
7	145	0.95	Elongation factor-1	1976
8	140	0.963	*Artemia salina* embryo	1975
9	134	0.987	DNA-dependent rna-polymerases	1977

**Table 8:** Top 10 popular keywords in titles, abstracts, and keywords in *Artemia* research in the world, based on the times cited in the WOS database.

Keyword	Times
Brine shrimp	946
Growth	768
Survival	496
*Artemia salina*	492
*Artemia*	448
Fish	414
Toxicity	397
Larvae	334
*Artemia franciscana*	320
Extract	304

**Table 9:** Top ten keywords with the strongest citation burst.

Keywords	Year	Strength	Begin	End	1970–2021
antimicrobial activity	1970	23.17	2011	2021	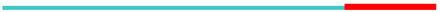
requirement	1970	19.58	1999	2008	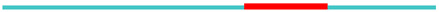
decapoda	1970	18.99	1997	2008	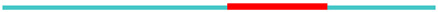
docosahexaenoic acid	1970	18.91	1996	2008	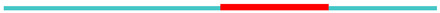
franciscana	1970	18.85	2004	2014	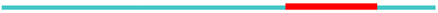
marine fish	1970	17.19	2000	2010	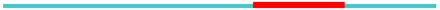
crustacea	1970	16.83	2003	2013	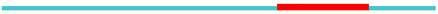
derivative	1970	15.77	2009	2018	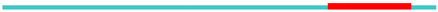
sparus aurata	1970	14.5	1996	2010	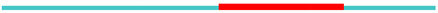
saccharomyces cerevisiae	1970	13.9	1991	2002